# Efficacy of the Tibial Transverse Bone Transport Technique in the Management of Thromboangiitis Obliterans: A Systematic Review

**DOI:** 10.3390/jcm15124521

**Published:** 2026-06-11

**Authors:** Ramy Samargandi, Mohammed R. Algethami

**Affiliations:** 1 Department of Surgery, College of Medicine, University of Jeddah, Jeddah 23218, Saudi Arabia; 2 Department of Family and Community Medicine, College of Medicine, University of Jeddah, Jeddah 23218, Saudi Arabia

**Keywords:** Buerger’s disease, thromboangiitis obliterans, distraction osteogenesis, transverse tibial transport, Ilizarov technique, limb salvage

## Abstract

**Background:** Thromboangiitis obliterans (TAO) is a nonatherosclerotic inflammatory vascular disorder affecting small- and medium-sized vessels, often leading to critical limb ischemia and a high risk of amputation. Conventional medical and surgical treatments remain limited, particularly for advanced diseases. Tibial transverse bone transport (TTT), based on the principles of distraction osteogenesis, has emerged as a novel technique to promote angiogenesis and improve microcirculation. This systematic review evaluated the clinical efficacy and safety of TTT in the management of TAO. **Methods:** A systematic review was conducted according to the PRISMA guidelines. A comprehensive search of PubMed, Scopus, Web of Science, ScienceDirect, and Google Scholar was performed until December 2025. Eligible studies included clinical investigations that evaluated TTT in patients with TAO. Data on patient characteristics, surgical techniques, clinical outcomes, and complications were extracted and analyzed descriptively because of the heterogeneity in study design and reporting. **Results:** Ten studies involving 368 patients were included in this review. TTT was consistently associated with significant clinical improvement, including pain relief, increased claudication distance, and ulcer healing, which were typically observed within weeks after the procedure. Limb salvage rates were high, with major amputation rates generally ranging from 3.3% to 13.3%. Objective improvements in perfusion parameters have also been reported in several studies. The most common complication was pin-site infection (up to 30%), while fractures, delayed consolidation, and osteomyelitis were less frequent complications. **Conclusions:** Current evidence suggests that TTT is a promising limb-salvage strategy for TAO and is associated with favorable clinical and functional outcomes, with an acceptable complication profile. However, the available evidence remains limited, partly because of the rarity of TAO and the specialized nature of the TTT procedure. Most available studies are observational, and further high-quality prospective and randomized trials are required to validate the long-term efficacy of this technique.

## 1. Introduction

Thromboangiitis obliterans (TAO), also known as Buerger’s disease, is a rare inflammatory vascular condition that primarily affects small-to medium-sized arteries and veins in the limbs [1,2]. It is strongly associated with tobacco use, especially smoking, and predominantly affects young male patients from regions such as the Mediterranean, the Middle East, and India [3,4]. Clinically, TAO commonly presents with distal ischemia, claudication, rest pain, ischemic ulceration, and a high risk of gangrene and limb loss. Without effective treatment, amputation rates in affected individuals can reach up to 75% within 3–10 years, imposing profound socioeconomic and psychological burdens [5]. This condition was initially referred to as endarteritis by Winiwarter [6,7]. Later, in 1908, Buerger named it TAO [8,9], after which the disease became widely recognized as Buerger’s disease. Despite its strong association with tobacco exposure, the exact etiology and pathophysiological mechanisms underlying TAO remain incompletely understood. Current evidence suggests that an abnormal inflammatory and immunologic response triggered by tobacco use, combined with possible genetic and environmental factors, may contribute to disease development [3,10].

The clinical management of Buerger’s disease has remained a challenge since its initial description. The mainstay of therapy is absolute smoking cessation, which remains the only consistently effective intervention shown to halt disease progression [11,12]. However, many patients continue to suffer from chronic ischemia despite cessation, prompting the exploration of additional therapeutic options [1,5]. A fully successful medical or surgical intervention has not been established, although various strategies have been attempted with partial benefits. Over the past two decades, medical, surgical, and endovascular therapies have included vasodilators, anticoagulants, prostanoids, sympathectomy, thrombolysis, arterial reconstruction, and bypass grafting [13,14,15]. Pharmacological approaches such as iloprost, clofibrate, cyclophosphamide, and calcium channel blockers have shown inconsistent improvements [3,5,16,17,18].

Recently, novel strategies, including spinal cord stimulation, angiogenic gene therapy, stem cell therapy, autologous bone marrow mononuclear cell implantation, and distraction osteogenesis through tibial transverse transport (TTT), have demonstrated potential in promoting neovascularization and limb salvage [19,20,21,22,23,24,25]. However, no single approach has emerged as the definitive treatment.

The achievement of neo-angiogenesis appears to hold the greatest promise, as chronic ischemia is the hallmark of TAO [26]. In this context, distraction osteogenesis, originally introduced by Ilizarov, has gained increasing attention. The method is based on the “law of tension-stress,” which postulates that gradual, controlled distraction stimulates the regeneration of bone and surrounding soft tissues, vascular proliferation, and the expression of angiogenic mediators such as vascular endothelial growth factor (VEGF), thereby promoting neovascularization and improved tissue perfusion [25,27]. Although initially developed for orthopedic reconstruction, subsequent observations have revealed improved local microcirculation and capillary network regeneration during tibial cortex transverse transport.

TTT has since been adopted as a surgical strategy for treating ischemic limb disease. By performing a corticotomy in the tibial cortex and applying controlled distraction via an external fixator, this technique stimulates angiogenesis, enhances blood and oxygen delivery to ischemic tissues, reduces ischemic pain, and lowers the risk of amputation [24,25,28]. Animal studies [27,29,30,31] and clinical investigations [25,32] have confirmed increased blood flow and microvascular proliferation following TTT.

Given these encouraging findings, this systematic review aimed to critically evaluate the efficacy and safety of tibial transverse bone transport in the clinical management of TAO. This review summarizes clinical outcomes, including ulcer healing, pain relief, limb salvage, and hemodynamic improvements, alongside surgical details such as corticotomy size, type of fixator, and distraction protocols. It also highlights the complications and knowledge gaps for future research.

## 2. Materials and Methods

### 2.1. Search Strategy

This systematic review was conducted in accordance with the Preferred Reporting Items for Systematic Reviews and Meta-Analyses (PRISMA) guidelines [33]. The review protocol was prospectively registered in the PROSPERO database (registration number: CRD420251160051). A comprehensive literature search was performed in PubMed, Scopus, Web of Science, ScienceDirect, Google Scholar, and China National Knowledge Infrastructure (CNKI) from database inception until 31 December 2025. The final electronic search was conducted on 31 December 2025. The PubMed search strategy combined Medical Subject Headings (MeSH) and free-text terms related to thromboangiitis obliterans and tibial transverse bone transport. The exact Boolean search syntax used in PubMed was as follows: (“Thromboangiitis obliterans” OR “Buerger disease”) AND (“tibial transverse transport” OR “transverse tibial transport” OR “transverse tibial corticotomy” OR “bone transport” OR “Ilizarov’s technique” OR “tibial cortex transverse transport” OR “cortex transverse transport” OR “distraction histogenesis” OR “distraction osteogenesis”). Equivalent search strategies adapted to database-specific indexing systems were applied to the remaining databases. No restrictions on publication year were applied during the initial search. No methodological filters for study design were used during database searching in order to maximize sensitivity. Only peer-reviewed studies with accessible full-text articles were considered eligible during the screening process. Google Scholar was used as a supplementary search source to identify additional potentially relevant studies through citation tracking and manual screening of references from eligible articles and narrative reviews. Gray literature was additionally explored through reference list screening and manual citation searching to minimize the risk of missing relevant studies. All retrieved records were exported into a reference management software program, and duplicate records were removed prior to screening. The detailed electronic search strategy for PubMed is provided in Supplementary Material S1.

### 2.2. Study Selection

The study selection process followed the PRISMA framework. After the removal of duplicates, two independent investigators screened all retrieved records based on their titles and abstracts. The full texts of potentially eligible articles were then independently assessed against predefined inclusion and exclusion criteria.

The inclusion criteria were defined to ensure the selection of clinically relevant and methodologically sound studies. Eligible articles were required to (i) focus specifically on the TTT technique in the management of TAO; (ii) be published in peer-reviewed journals with an accessible English full-text version; (iii) include clinical study designs such as randomized controlled trials (RCTs), non-randomized comparative studies, prospective or retrospective cohort studies, or case series; and (iv) provide sufficient clinical details, including sample size, patient characteristics, follow-up duration, details of the surgical technique, and postoperative outcomes and complications.

Studies were excluded if they were case reports, conference abstracts, expert consensus documents, or investigations involving patients younger than 18 years. Articles were also excluded if they failed to report essential variables, such as sample size, demographic details, type of TTT surgery, follow-up period, or treatment outcomes. Non-English studies were initially screened when identified through database or reference searches; however, studies without accessible English versions were not included in the final qualitative synthesis. Following this process, 10 studies met the inclusion criteria and were included in the qualitative synthesis.

### 2.3. Data Extraction

Full-text articles meeting the eligibility criteria were retrieved for a detailed review. Two independent investigators extracted the data from each study. The information collected included bibliographic details (first author, title, year of publication, country, and study design), clinical data (sample size, patient demographics, and follow-up duration), and details of the surgical technique (type of corticotomy, fixation method, and distraction protocol). The clinical outcomes of interest included pain relief, improvement in claudication distance, ulcer healing, and overall clinical success. Objective parameters, when available, such as hemodynamic and angiographic findings, were recorded. Complication profiles were systematically extracted, including major outcomes such as amputation rates and procedure-related complications, including pin-site infection, fracture, delayed consolidation, osteomyelitis, and need for reintervention. Any discrepancies between the investigators were resolved through discussion and consensus agreement.

### 2.4. Quality Assessment and Statistical Analysis

The included studies comprised retrospective and prospective cohort studies, case series, comparative observational studies, and one randomized controlled trial. As most studies were non-randomized, the methodological quality of the non-randomized studies was assessed using the Risk of Bias in Non-randomized Studies of Interventions (ROBINS-I) tool [34]. This tool evaluates seven domains through which bias may be introduced, including bias due to confounding, selection of participants, classification of interventions, deviations from intended interventions, missing data, measurement of outcomes, and selection of the reported result. Each domain was categorized as low, moderate, serious, or critical risk of bias, and an overall judgment was subsequently assigned for each study. The randomized controlled trial by Zuo et al. [35] was assessed separately using the revised Cochrane Risk of Bias tool for randomized trials (ROB 2) [36]. This tool evaluates five domains, including bias arising from the randomization process, deviations from intended interventions, missing outcome data, measurement of outcomes, and selection of the reported result. Each domain was judged as low risk, some concerns, or high risk of bias, and an overall risk-of-bias judgment was subsequently determined. The results of the ROBINS-I assessment were visualized through both ‘traffic light’ and bar plots generated using the robvis web application (https://mcguinlu.shinyapps.io/robvis/, accessed on 11 May 2026) [37]. while the ROB 2 assessment was presented separately.

### 2.5. Statistical Analysis

Descriptive statistics were used to summarize patient demographics, clinical outcomes, and procedural details. Continuous variables, such as age and follow-up duration, were expressed as means with ranges. Clinical outcomes, including success rates and complication profiles (infection, fracture, and revision procedures), were reported as proportions relative to the total population. Due to heterogeneity in study design, outcome definitions, follow-up duration, reporting methodology, and complication reporting, formal quantitative synthesis through meta-analysis was deemed inappropriate, as this could have limited the validity and interpretability of pooled estimates; therefore, the findings were summarized descriptively despite partial overlap in outcomes such as limb salvage, ulcer healing, and pain improvement across several studies.

## 3. Results

### 3.1. Study Selection

**Figure 1:**
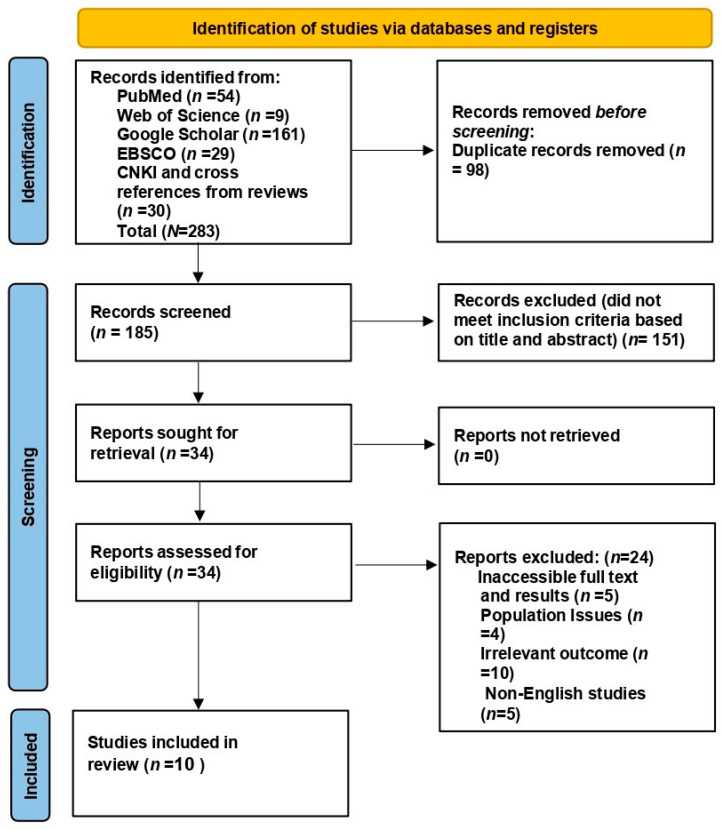
Schematic of the PRISMA methodology followed in this systematic review. CNKI: China National Knowledge Infrastructure.

A total of 283 records were identified in the initial database search. After the removal of duplicates and screening of titles and abstracts, potentially relevant full-text articles were assessed for eligibility. Ten studies met the predefined inclusion criteria and were included in the final qualitative synthesis [25,26,35,38,39,40,41,42,43,44] (Figure 1).

### 3.2. Study Characteristics

The included studies were published between 2011 and 2025 and were predominantly conducted in India, Bangladesh, and China. The study designs comprised retrospective cohort studies, prospective interventional studies, case series, and comparative observational studies. A total of 368 patients underwent TTT for TAO. The sample sizes ranged from 5 to 64 patients. The mean patient age, when reported, ranged from 35 to 53.2 years, while the follow-up duration varied from 1 month to more than 5 years (Table 1).

### 3.3. Surgical Technique and Procedural Characteristics

Despite minor technical variations, the operative principles were largely consistent throughout the studies. Most authors described corticotomy over the middle third of the tibia, commonly using an anterolateral approach, followed by gradual transverse distraction using an Ilizarov ring external fixator. More recent studies by Zhao et al. [25] and Hong et al. [42] utilized monolateral external fixation systems. The corticotomy dimensions generally ranged from 10 to 12 cm in length and 1.5 to 3 cm in width.

A latency period of approximately 6–10 days before distraction has been commonly reported. In most studies, distraction was performed at approximately 1 mm/day, often divided into 0.25 mm increments every 6 h, until a transport distance of approximately 2–2.5 cm was achieved [38,39,40]. Although corticotomy dimensions varied slightly between studies, none of the included investigations performed a formal comparative analysis evaluating the relationship between osteotomy size and clinical outcomes.

### 3.4. Clinical Outcomes

Overall, all included studies reported favorable clinical improvement following TTT, although outcome definitions differed substantially across studies, precluding formal pooled efficacy analysis (Table 2).

#### 3.4.1. Pain Relief

Pain reduction was one of the most consistently reported outcomes. Kulkarni et al. [39] reported complete pain relief in the majority of patients, whereas Rohit and Verma [26] found that most patients became pain-free following treatment. Chouhan et al. [40] demonstrated marked postoperative reductions in pain scores during follow-up. Patwa and Krishnan [38] and Islam et al. [44] also described the early disappearance of rest pain in most treated patients.

#### 3.4.2. Claudication Distance and Functional Recovery

Improvements in walking tolerance and claudication distance were frequently observed. Chouhan et al. [40] reported progressive postoperative gains in walking distance, while Nesari and Wali [41] described postoperative ambulation exceeding 1.5 km. Hong et al. [42] reported superior functional recovery and quality-of-life outcomes compared with conventional management.

#### 3.4.3. Hemodynamic and Angiographic Outcomes

Beyond subjective symptom relief, several studies have reported objective improvements in vascular perfusion following TTT. Chouhan et al. [40] described postoperative computed tomography angiographic evidence of improved vascularity in a subset of patients during follow-up. Hong et al. [42] reported significant postoperative improvements in hemorheological parameters and vascular-related clinical indices compared with conventional treatment. Nesari and Wali [41] noted postoperative improvements in limb perfusion and oxygenation parameters. In a comparative study by Zuo et al. [35], TTT was associated with increased arterial diameter, enhanced regional blood flow, higher dorsal foot skin temperature, and increased vascular endothelial growth factor expression, supporting a potential angiogenic mechanism.

#### 3.4.4. Ulcer Healing and Tissue Recovery

Ulcer healing is commonly achieved in patients with ischemic wounds. Kulkarni et al. [39] reported the initiation of ulcer healing within 4–6 weeks. Zhao et al. [25] found that most wounds healed during the first treatment cycle, with complete healing after two treatment cycles. Patwa and Krishnan [38], Bari et al. [43], and Islam et al. [44] also described the healing of trophic ulcers and stump wounds and improvement in skin temperature, color, and local perfusion.

### 3.5. Overall Clinical Success

Reported favorable outcome rates were consistently high, although they were based on heterogeneous scoring systems. Studies using excellent/good grading systems generally reported success rates between 80% and 88%, whereas studies describing global improvement or limb salvage reported rates approaching 90% to100% in selected cohorts [38,40,41,42]. Only Patwa et al. [38] and Islam et al. [44] separately reported early and late outcomes, demonstrating a decline from 93.3% to 80% and from 90.3% to 80%, respectively, during longer-term follow-up. However, interpretation of the durability of these outcomes should be approached cautiously because follow-up duration varied substantially across studies, ranging from 1 month to more than 5 years.

### 3.6. Limb Salvage and Amputation

Major amputation remains necessary in a minority of patients, typically in the setting of advanced gangrene, severe infection, or progression despite treatment. Across studies reporting this outcome, below-knee amputation rates generally ranged from 3.3% to 13.3%, suggesting favorable limb preservation in appropriately selected patients [25,38,39,40]. Although smoking cessation was consistently emphasized as a fundamental component of TAO management, most studies did not formally stratify limb salvage or amputation outcomes according to postoperative smoking status, limiting assessment of its direct influence on surgical outcomes.

### 3.7. Complications

The most frequently reported complication was pin-site or pin-tract infection, with rates ranging from 16% to 30% in studies that specifically documented this outcome. These events were generally minor and were successfully managed with dressings, local care, and oral antibiotics [26,38,40].

Fractures during the consolidation phase or after fixator removal were less common, with reported rates ranging from 3.3% to 8%. Delayed regenerate consolidation was reported as an isolated event by Rohit et al. [26]. Osteomyelitis occurred in the cohort of Kulkarni et al. [39], and was successfully managed after debridement. Additional isolated complications included stitch necrosis and protrusion of the transported bone segment, requiring secondary intervention.

### 3.8. Quality Assessment

**Figure 2:**
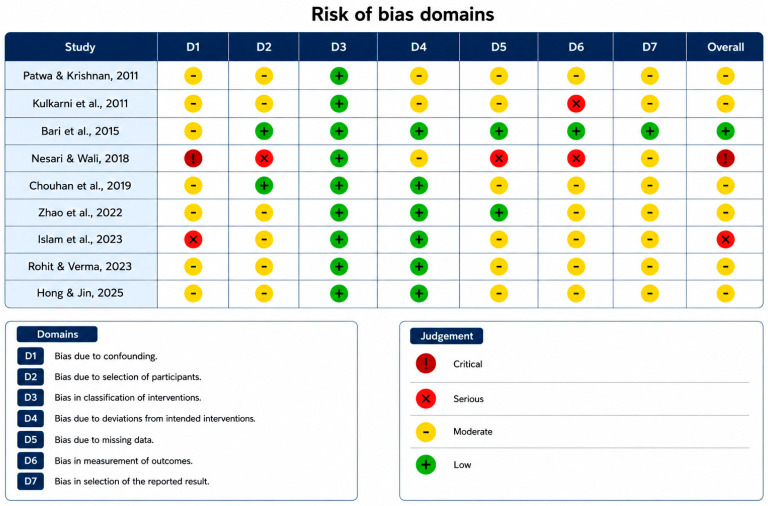
Traffic light plot summarizing the ROBINS-I risk-of-bias assessment across the included non-randomized studies [25,26,38,39,40,41,42,43,44].

**Figure 3:**
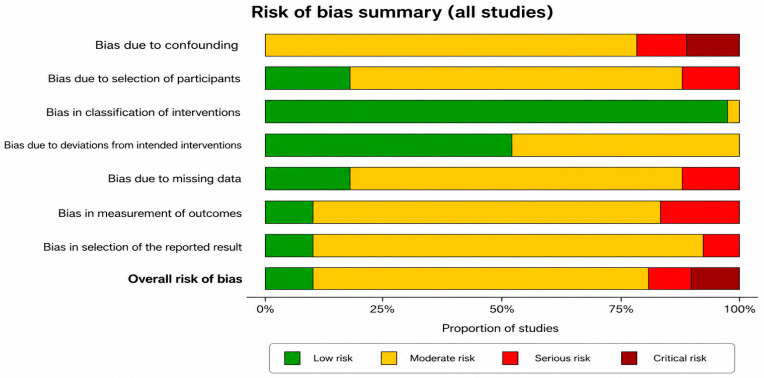
Summary bar plot representing the ROBINS-I risk-of-bias analysis for the included non-randomized studies.

**Figure 4:**
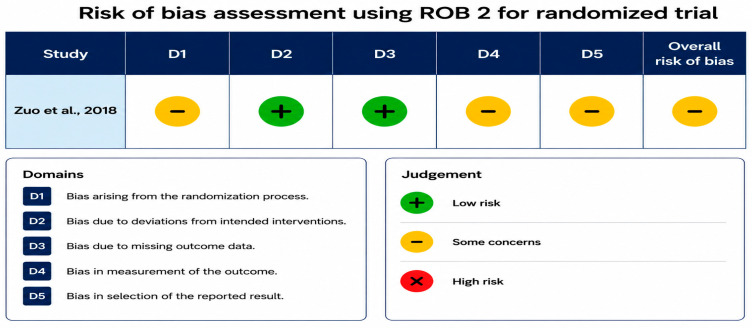
ROB 2 risk-of-bias assessment of the randomized controlled trial by Zuo et al. (2018) [35].

Risk-of-bias assessment using the ROBINS-I tool demonstrated an overall moderate-to-serious risk of bias across the nine non-randomized studies (Figure 2 and Figure 3). The principal methodological limitations included retrospective study designs, small sample sizes, absence of blinding, heterogeneous outcome definitions, and limited adjustment for confounding variables. The randomized controlled trial assessed using ROB 2 demonstrated some concerns regarding risk of bias, mainly related to the randomization process and outcome assessment (Figure 4). Nevertheless, the available literature consistently demonstrated favorable trends in pain relief, ulcer healing, functional recovery, objective perfusion improvement, and limb preservation following TTT.

## 4. Discussion

This systematic review demonstrates that TTT is consistently associated with favorable clinical outcomes in patients with TAO, including significant pain relief, improvement in claudication distance, ulcer healing, and high rates of limb salvage. Despite the heterogeneity in study design and outcome reporting, these findings were reproducible across all included studies, suggesting a potentially beneficial therapeutic effect of the technique in patients with advanced ischemic disease who are often refractory to conventional treatments.

The biological rationale for TTT is based on the principles of distraction osteogenesis described by Ilizarov [27], whereby gradual mechanical distraction induces not only bone regeneration but also adaptive changes in the surrounding soft tissues, including vascular structures. Experimental and clinical studies have shown that this process is associated with the stimulation of angiogenesis and improvement in microcirculation, mediated in part by the upregulation of factors such as VEGF and hypoxia-inducible pathways [45,46,47,48,49,50]. These mechanisms provide a plausible explanation for the consistent improvements in perfusion-related outcomes observed in the included studies.

The clinical use of transverse bone transport in ischemic limb disease can be traced back to the early work by Fokin et al. [51,52,53], who reported the application of TTT in patients with chronic limb ischemia, demonstrating improvement in pain and tissue viability. This approach was further developed by Shevtsov et al. [54,55], who reported a large clinical series of patients with obliterative vascular diseases, including TAO. In these studies, TTT was associated with improved limb perfusion, collateral vessel formation, ulcer healing, and reduced amputation rates. Although these early reports lacked standardized methodologies and modern imaging assessments, their findings are broadly consistent with contemporary studies and support the reproducibility of the technique across different clinical settings.

The findings of the present review are consistent with these earlier observations. Across the included studies, TTT was associated with improvement in pain, ulcer healing, and limb salvage in patients with advanced TAO. These favorable outcomes likely reflect enhanced microvascular perfusion induced by distraction-mediated angiogenesis. However, postoperative inflammatory markers such as CRP or ESR were not systematically evaluated, limiting assessment of the potential interaction between revascularization and inflammatory activity in TAO.

Similar outcomes have been reported for other ischemic conditions, particularly diabetic foot ulcers (DFUs). In a large prospective multicenter cohort study, Chen et al. [56] reported ulcer healing in 94.9% of patients, with major amputation and recurrence rates of 4.9% and 3.1%, respectively, at the 2-year follow-up. These findings are further supported by the systematic review and meta-analysis by Hu et al. [57], which demonstrated pooled healing rates of approximately 96% and limb salvage rates approaching 98%, along with significant improvements in perfusion parameters. More recently, Luxon et al. [58] systematically reviewed the application of TTT in DFUs and analyzed 724 patients treated with this technique, similarly reporting favorable outcomes regarding revascularization, wound healing, pain reduction, and limb salvage, with relatively low complication rates. The consistency of outcomes between TAO and DFU suggests that the therapeutic effect of TTT is primarily related to its ability to enhance microcirculation and promote angiogenesis, rather than disease-specific mechanisms. These modern findings are also broadly consistent with earlier clinical experiences reported by Fokin and Shevtsov [51,52,54,55] regarding the application of distraction osteogenesis techniques in chronic limb ischemia and obliterative vascular diseases.

Alternative anatomical approaches to transverse bone transport further support the generalizability of this technique in the field. Chang et al. [59] demonstrated that iliac crest bone transport could achieve comparable improvements in pain, ulcer healing, and vascular parameters in patients with TAO, suggesting that the angiogenic effect of distraction osteogenesis is not limited to the tibia. Similarly, Mo et al. [60] reported the successful application of transverse cortex transport in the ulna for upper limb ischemic conditions, with significant improvements in pain, ulcer healing, and vascularity in the patients. These findings indicate that the biological response to controlled mechanical distraction may be applicable to different anatomical regions.

The complication profile of TTT should also be considered. Pin-site infection was the most commonly reported complication, with rates varying widely across studies, although most cases were minor and resolved with local care and antibiotics [26,38,40]. Fractures at the corticotomy site were less frequent and generally manageable, often healing with conservative treatment or temporary stabilization [38,40]. Delayed consolidation and osteomyelitis have rarely been reported but remain important considerations, particularly in earlier studies [39]. Overall, the complication profile appears acceptable when balanced against the potential benefits of limb salvage, especially in patients with otherwise limited treatment options.

This review has several limitations. First, the available literature consisted predominantly of observational studies, with only one small randomized controlled trial identified, thereby limiting the overall level of evidence. In addition, quality assessment demonstrated moderate-to-serious risk of bias across several included studies. Second, there was considerable heterogeneity in study design, patient selection, surgical technique, outcome definitions, follow-up duration, and reporting methodology, which precluded formal meta-analysis and limited direct comparisons between studies. Third, most studies originated from a limited number of geographic regions, potentially affecting their generalizability. Fourth, outcome assessment lacked standardization across studies. Definitions of clinical improvement and outcomes varied considerably, while validated vascular classification systems or chronic limb-threatening ischemia staging were not reported or utilized across the included studies. Blinded outcome assessment was also not reported in the available literature. In addition, several vascular-specific factors that may influence treatment outcomes, including smoking status during follow-up, distal runoff anatomy, concomitant pharmacologic therapy, and prior sympathectomy, were generally not reported in the available literature. Furthermore, diagnostic methodology for TAO was not uniformly described across studies, and retrospective cohorts may carry a risk of overlap or misclassification with atherosclerotic chronic limb-threatening ischemia. Fifth, language restriction may have introduced selection bias, particularly because part of the literature originates from Chinese and Russian publications, and studies without accessible English versions were not included in the current systematic review. Finally, follow-up duration varied substantially across studies, and only a limited number of studies provided separate early and long-term outcome assessment, restricting definitive evaluation of the durability of clinical benefit and late complications.

## 5. Conclusions

TTT represents a biologically plausible and potentially beneficial approach for the management of TAO, with consistent evidence supporting improvements in pain, ulcer healing, and limb salvage. Its mechanism, based on the enhancement of microcirculation through distraction-induced angiogenesis, appears to be applicable across a range of ischemic conditions. However, further high-quality prospective randomized studies using standardized diagnostic criteria, validated vascular outcome measures, and longer-term follow-up are required to confirm these findings and establish reproducible treatment protocols.

## Figures and Tables

**Table 1:** Characteristics and surgical details of studies evaluating transverse tibial bone transport for thromboangiitis obliterans.

Author (Year)	Country	Study Type	Number of Cases	Mean Age in Years	Fixator Type	Osteotomy Site	Osteotomy Size	Distraction Duration (Latency + Distraction Time)
**Patwa et al.** [38]	India	Retrospective	60	43.22 ± 5	Ring EF	Anterolateral Middle 1/3 of tibia	NR	7 days latency + 20 days distraction (0.25 mm every 6 h → 2 cm)
**Kulkarni et al.** [39]	India	Retrospective	30	35 (25–50)	Ring EF	Anterolateral Middle 1/3 of tibia	12 × 3 cm	10 days latency + 20 days distraction (1 mm/day → 2 cm)
**Chouhan et al.** [40]	India	Prospective	50	44 (35–55)	Ring EF	Anterolateral Middle 1/3 of tibia	NR	10 days latency + 6 weeks distraction (0.5 mm/day)
**Rohit Verma et al.** [26]	India	Case series	10	42 (34–70)	Ring EF	Anterolateral Middle 1/3 of tibia	12 cm	10 days latency + 25 days distraction (0.25 mm × 4/day → 2.5 cm)
**Islam et al.** [44]	Bangladesh	Prospective	60	39	Ring EF	Anterolateral Middle 1/3 of tibia	NR	7 days latency + 20 days distraction (0.25 mm every 6 h → 2 cm)
**Bari** [43]	Bangladesh	Retrospective	18	NR	Ring EF	Anterolateral Middle 1/3 of tibia	12 × 2 cm	Latency NR + 3 weeks distraction (1 mm/day × 3 weeks)
**Nesari & Wali** [41]	India	Case series	5	43	Ring EF	Anteromedial Middle 1/3 of tibia	12–15 cm	7 days latency + distraction (0.25 mm every 6 h → 2–2.5 cm)
**Zhao et al.** [25]	China	Case series	26	40	monolateral EF	Anterolateral Middle 1/3 of tibia	10 × 2 cm	One treatment cycle = 20 days
**Zuo et al.** [35]	China	Randomized study (TTT vs. PTBA)	*n* = 90 (45/45)	53.2	Ring EF	Anterolateral Middle 1/3 of tibia	10–12 cm × 1.5–2 cm	NR
**Hong et al.** [42]	China	Retrospective comparative cohort	*n* = 119 (TTT = 64; control = 55)	49.3	monolateral EF	Anteromedial Middle 1/3 of tibia	10 × 2 cm	6 days latency + 0.33 mm/8 h (1 mm/day) for 2 weeks

EF: external fixator; NR: not reported; PTBA: percutaneous transluminal balloon angioplasty; TTT: transverse tibial bone transport.

**Table 2:** Clinical outcomes, efficacy, complications, and conclusions of the studies.

Author (Year)	Follow Up	Notable Outcomes	Efficacy (%)	Complications (n, %)	Author Conclusion
**Patwa et al.** [38]	5.4 years	96% had disappearance of rest pain + improved warmth/color + wound/ulcer healing	Early excellent-good: 93.3%; late excellent-good: 80%	BKA: 2/60 (3.3%); protruded bone block needing removal: 4/60 (6.7%); fracture: 2/60 (3.3%); Pin tract infection 18/60 (30%); stich necrosis 10/60 (16.6%)	Low-cost, technically simple; “excellent results” in grade III–IV
**Kulkarni et al.** [39]	4.5 years	Pain-free: 25/30; partial relief: 1/30; ulcers started healing by 4–6 weeks	Excellent-good: 83.3%	BKA: 4/30 (13.3%) osteomyelitis: 2/30 (6.7%)	TTT can be used for TAO with acceptable complication rate
**Chouhan et al.** [40]	12.5 months	VAS improved markedly; rest pain/claudication distance improved over follow-up	Excellent-good: 88%	Pin-tract infection: 8/50 (16%); fracture: 4/50 (8%); BKA: 6 (12%)	TTT is a reliable, cost-effective limb salvage option for TAO patients who fail other treatments Smoking cessation is critical for success
**Rohit Verma et al.** [26]	12 months	Claudication distance improved over follow-up; 8/10 pain-free	Excellent-good: 80%	Pin-site infection: 3/10 (30%); delayed consolidation: 1/10 (10%); BKA: 1/10 (10%)	TTT is a reliable, cost-effective option to improve limb ischemia and help limb salvage in TAO
**Islam et al.** [44]	5.4 years	Rest pain disappeared within 2–4 weeks in most; analgesia reduced within 2 days; improved warmth/color by ~10 days; healing signs by ~2 weeks	Early excellent-good: 90.3%; late excellent-good: 80%	BKA: 2/60 (3.3%)	TTT is effective and cost-efficient for TAO; emphasizes smoking cessation and long-term follow-up
**Bari** [43]	5 years	Clinical improvement within weeks; arteriogram improved at 2 months	Excellent-good: 83.3%	BKA: 2/18 (11.1%)	TTT is an effective limb-salvage technique in TAO that may prevent amputation.
**Nesari & Wali** [41]	33 months	Pain reduced; dramatic improvement in claudication; skin ischemic changes improved; SpO_2_ > 95% at removal; walking > 1.5 km	Clinical improvement: 100%	NR	TTT is a good, cost-effective alternative that may avoid higher-level amputations when combined with tobacco cessation
**Zhao et al.** [25]	14.2 months	24/26 limbs successfully treated; rest pain and ischemia improved; wounds healed within 1–2 “cycles”	Limb salvage 92.3%	BKA: 2/26 (7.7%)	TTT is worth considering for TAO-related refractory ulcers
**Zuo et al.** [35]	1 months	Faster wound healing and pain disappearance vs. PTBA; improved claudication/rest pain/ulcer-gangrene improvement counts reported	Claudication: 86.7%; rest pain: 97.8%; ulcer/gangrene improvement: 84.4%	NR	TTT achieves better outcomes than PTBA for chronic lower limb ischemic disease
**Hong et al.** [42]	6 months	Significant improvement in ABI, walking distance, pain, hemorheology, and QoL compared to the control.	TTT: 89.06% vs. Control: 70.91%	NR	TTT provides superior therapeutic benefits and is an effective treatment for TAO

ABI: Ankle-brachial index; BKA: below-knee amputation; NR: not reported; QoL: quality of life; TTT: transverse tibial bone transport; TAO: Thromboangiitis obliterans; VAS: visual analog scale; PTBA: percutaneous transluminal balloon angioplasty.

## Data Availability

The data presented in this study are available upon request from the corresponding author.

## Supplementary Materials

The following supporting information can be downloaded at: https://www.mdpi.com/article/10.3390/jcm15124521/s1.

## References

[1] Olin J.W., Shih A. (2006). Thromboangiitis Obliterans (Buerger’s Disease). Curr. Opin. Rheumatol..

[2] Fiessinger J.-N., Frank M. (2015). Thromboangiitis Obliterans (Buerger’s Disease). Rev. Prat..

[3] Vijayakumar A., Tiwari R., Kumar Prabhuswamy V. (2013). Thromboangiitis Obliterans (Buerger’s Disease)—Current Practices. Int. J. Inflamm..

[4] Arkkila P.E. (2006). Thromboangiitis Obliterans (Buerger’s Disease). Orphanet J. Rare Dis..

[5] Piazza G., Creager M.A. (2010). Thromboangiitis Obliterans. Circulation.

[6] Winiwarter F. (1879). On a Peculiar Form of Endarteritis and Endophlebitis with Gangrene of the Foot. Arch. Klin. Chir..

[7] Herrington J.L., Grossman L.A. (1968). Surgical Lesions of the Small and Large Intestine Resulting from Buerger’s Disease. Ann. Surg..

[8] Buerger L. (1952). Thrombo-Angitis Obliterans; a Study of the Vascular Lesions Leading to Presenile Spontaneous Gangrene. Am. J. Med..

[9] Rivera-Chavarría I.J., Brenes-Gutiérrez J.D. (2016). Thromboangiitis Obliterans (Buerger’s Disease). Ann. Med. Surg..

[10] Klein-Weigel P.F., Richter J.G. (2014). Thromboangiitis Obliterans (Buerger’s Disease). VASA Z. Gefasskrankh..

[11] Qaja E., Muco E., Hashmi M.F. (2026). Buerger Disease. StatPearls.

[12] Gundogmus C.A., Samadli V., Sorkun M., Oguzkurt L. (2023). The Effect of Smoking Cessation on the Technical Success of Endovascular Treatment for Thromboangiitis Obliterans. J. Vasc. Interv. Radiol. JVIR.

[13] Zheng G., Xie H., Lai M., Liu X. (2024). Short-Term Efficacy of Endovascular Procedures for Lower Extremity Thromboangiitis Obliterans (Buerger’s Disease). Postgrad. Med..

[14] Salimi J., Cheraghali R., Omrani Z., Farshidmehr P., Afghani R. (2022). Surgical Treatment Options for Buerger’s Disease (Experience with 315 Cases in Iran). Med. J. Islam. Repub. Iran.

[15] Shekouhi R., Mumtaz M., Naqvi H., Azizi A., Crawford K.M., Jacobs B.N., Chim H. (2025). Treatment Options for Buerger Disease: A Systematic Review and Meta-Analysis of Outcomes. J. Surg. Res..

[16] Cacione D.G., Macedo C.R., do Carmo Novaes F., Baptista-Silva J.C. (2020). Pharmacological Treatment for Buerger’s Disease. Cochrane Database Syst. Rev..

[17] Bozkurt A.K., Cengiz K., Arslan C., Mine D.Y., Oner S., Deniz D.B., Ufuk D. (2013). A Stable Prostacyclin Analogue (Iloprost) in the Treatment of Buerger’s Disease: A Prospective Analysis of 150 Patients. Ann. Thorac. Cardiovasc. Surg. Off. J. Assoc. Thorac. Cardiovasc. Surg. Asia.

[18] Saha K., Chabra N., Gulati S.M. (2001). Treatment of Patients with Thromboangiitis Obliterans with Cyclophosphamide. Angiology.

[19] Cacione D.G., do Carmo Novaes F., Moreno D.H. (2018). Stem Cell Therapy for Treatment of Thromboangiitis Obliterans (Buerger’s Disease). Cochrane Database Syst. Rev..

[20] Fabregat G., Villanueva V.L., Asensio J.M., De Andrés J., López D. (2011). Spinal Cord Stimulation for the Treatment of Buerger Disease: A Report on 3 Cases. Clin. J. Pain.

[21] Barć P., Lubieniecki P., Antkiewicz M., Kupczyńska D., Barć J., Frączkowska-Sioma K., Dawiskiba T., Dorobisz T., Sekula W., Czuwara B. (2024). Gene Therapy of Thromboangiitis Obliterans with Growth Factor Plasmid (VEGF165) and Autologous Bone Marrow Cells. Biomedicines.

[22] Saito S., Nishikawa K., Obata H., Goto F. (2007). Autologous Bone Marrow Transplantation and Hyperbaric Oxygen Therapy for Patients with Thromboangiitis Obliterans. Angiology.

[23] Donas K.P., Schulte S., Ktenidis K., Horsch S. (2005). The Role of Epidural Spinal Cord Stimulation in the Treatment of Buerger’s Disease. J. Vasc. Surg..

[24] Liu G., Li S., Kuang X., Zhou J., Zhong Z., Ding Y., Lu W., Zhao J., Chen Y., Hua Q. (2020). The Emerging Role of Tibial Cortex Transverse Transport in the Treatment of Chronic Limb Ischemic Diseases. J. Orthop. Transl..

[25] Zhao L., Lei Y., Pang M., Wei Z. (2022). An Improved Bone Transport Surgical Method for Treating Chronic Ischemic Ulcers (Thromboangiitis Obliterans). Front. Surg..

[26] Rohit K., Verma V. (2023). Management of Buerger’s Disease (Thromboangiitis Obliterans) of the Lower Limb by Horizontal Distraction and Corticotomy by Ilizarov’s Technique. J. Orthop. Dis. Traumatol..

[27] Ilizarov G.A. (1989). The Tension-Stress Effect on the Genesis and Growth of Tissues. Part I. The Influence of Stability of Fixation and Soft-Tissue Preservation. Clin. Orthop..

[28] Liu Z., Xu C., Yu Y.-K., Tu D.-P., Peng Y., Zhang B. (2022). Twenty Years Development of Tibial Cortex Transverse Transport Surgery in PR China. Orthop. Surg..

[29] Tian W., Zhang L., Wang Y., Lin L., Jiang W., Dai G., Feng B. (2024). Tibial Transverse Transport Promotes Wound Healing in Diabetic Foot Ulcers by Stimulating Endothelial Progenitor Cell Mobilization and Homing Mediated Neovascularization. Ann. Med..

[30] Qin W., Liu K., Su H., Hou J., Yang S., Pan K., Yang S., Liu J., Zhou P., Lin Z. (2024). Tibial Cortex Transverse Transport Promotes Ischemic Diabetic Foot Ulcer Healing via Enhanced Angiogenesis and Inflammation Modulation in a Novel Rat Model. Eur. J. Med. Res..

[31] Yang Y., Li Y., Pan Q., Bai S., Wang H., Pan X., Ling K.-K., Li G. (2022). Tibial Cortex Transverse Transport Accelerates Wound Healing via Enhanced Angiogenesis and Immunomodulation. Bone Jt. Res..

[32] Nie X., Kuang X., Liu G., Zhong Z., Ding Y., Yu J., Liu J., Li S., He L., Su H. (2020). Tibial Cortex Transverse Transport Facilitating Healing in Patients with Recalcitrant Non-Diabetic Leg Ulcers. J. Orthop. Transl..

[33] Page M.J., McKenzie J.E., Bossuyt P.M., Boutron I., Hoffmann T.C., Mulrow C.D., Shamseer L., Tetzlaff J.M., Akl E.A., Brennan S.E. (2021). The PRISMA 2020 Statement: An Updated Guideline for Reporting Systematic Reviews. BMJ.

[34] Sterne J.A., Hernán M.A., Reeves B.C., Savović J., Berkman N.D., Viswanathan M., Henry D., Altman D.G., Ansari M.T., Boutron I. (2016). ROBINS-I: A Tool for Assessing Risk of Bias in Non-Randomised Studies of Interventions. BMJ.

[35] Zuo Q., Gao F., Song H., Zhou J. (2018). Application of Ilizarov Transverse Tibial Bone Transport and Microcirculation Reconstruction in the Treatment of Chronic Ischemic Diseases in Lower Limbs. Exp. Ther. Med..

[36] Sterne J.A.C., Savović J., Page M.J., Elbers R.G., Blencowe N.S., Boutron I., Cates C.J., Cheng H.-Y., Corbett M.S., Eldridge S.M. (2019). RoB 2: A Revised Tool for Assessing Risk of Bias in Randomised Trials. BMJ.

[37] McGuinness L.A., Higgins J.P.T. (2021). Risk-of-Bias VISualization (Robvis): An R Package and Shiny Web App for Visualizing Risk-of-Bias Assessments. Res. Synth. Methods.

[38] Patwa J.J., Krishnan A. (2011). Buerger’s Disease (Thromboangiitis Obliterans)-Management by Ilizarov’s Technique of Horizontal Distraction. A Retrospective Study of 60 Cases. Indian J. Surg..

[39] Kulkarni S., Kulkarni G., Shyam A.K., Kulkarni M., Kulkarni R., Kulkarni V. (2011). Management of Thromboangiitis Obliterans Using Distraction Osteogenesis: A Retrospective Study. Indian J. Orthop..

[40] Chouhan A., Meena D.S., Meena U.K., Behera P., Yadav L., Gupta V. (2019). Limb Salvage in Buerger’s Disease by Distraction Histogenesis: A Prospective Study with Literature Review. J. Clin. Orthop. Trauma.

[41] Nesari S.S., Wali P.C. (2018). Management of Thromboangitis Obliterans by Ilizarov Technique. MedPulse Int. J. Orthop..

[42] Hong M., Jin Q. (2025). Therapeutic Effects of Transverse Tibial Bone Transport in Lower Limb Thromboangiitis Obliterans. Am. J. Transl. Res..

[43] Bari M.M., Shahidul I., Rouf A.H. (2015). Management of Buerger’s Disease (TAO) With Ilizarov (A Retrospective Study of 18 Cases). MOJ Orthop. Rheumatol..

[44] Islam D.K., Chowdhury S., Forhad S., Siddiqui E.H., Majid M.A., Rahman M.M. (2023). Buerger’s Disease (Thromboangiitis Obliterans)-Management by Ilizarov’s Technique of Horizontal Distraction—A Prospective Interventional Study.

[45] Schroeder Q.M., Paredes D.U., Montion M., Powell E.R., Topp D.R.C., Golshteyn G., Oji E., Wilkinson K.A., Tran D.V., Smith K.M. (2025). Tibial Cortex Transverse Transport: Historical Evolution, Clinical Applications, and Future Directions. Foot Ankle Surg. Tech. Rep. Cases.

[46] Liao M.-M., Zhang F., Wang Y.-K., Wang M.-W., Cao J.-R., Jin Z.-H., Ren Y.-J., Chen S. (2026). Transverse Tibial Bone Transport Promotes Distraction Osteogenesis and Improves Blood Flow in the Management of Diabetic Foot. World J. Diabetes.

[47] Qin Q., Liu Y., Yang Z., Aimaijiang M., Ma R., Yang Y., Zhang Y., Zhou Y. (2022). Hypoxia-Inducible Factors Signaling in Osteogenesis and Skeletal Repair. Int. J. Mol. Sci..

[48] Wan C., Gilbert S.R., Wang Y., Cao X., Shen X., Ramaswamy G., Jacobsen K.A., Alaql Z.S., Eberhardt A.W., Gerstenfeld L.C. (2008). Activation of the Hypoxia-Inducible Factor-1α Pathway Accelerates Bone Regeneration. Proc. Natl. Acad. Sci. USA.

[49] Ou S., Xu C., Li G., Sun H., Yang Y., Lu H., Li W., Qi Y. (2020). Effect of transverse tibial bone transport on expression of serum angiogenesis-related growth factors. Zhongguo Xiu Fu Chong Jian Wai Ke Za Zhi Zhongguo Xiufu Chongjian Waike Zazhi Chin. J. Reparative Reconstr. Surg..

[50] Ou S., Xu C., Yang Y., Chen Y., Li W., Lu H., Li G., Sun H., Qi Y. (2022). Transverse Tibial Bone Transport Enhances Distraction Osteogenesis and Vascularization in the Treatment of Diabetic Foot. Orthop. Surg..

[51] Fokin A.A., Fokin A.A., Verbovetskiĭ L.P. (1993). Short and long-term results of non-standard revascularization of the lower extremities. Grud. Serdechnososudistaia Khir..

[52] Fokin A.A., Verbovetskiĭ L.P., Fokin A.A., Kulak A.N. (1990). The effectiveness of G. A. Ilizarov’s method in treating patients with III- and IV-stage chronic ischemia of the lower extremities. Vestn. Khir. Im. II Grek..

[53] Fokin A.A., Kulak A.N., Fokin A.A., Verbovetskiĭ L.P. (1988). Treatment of occlusive diseases of the arteries of the lower extremities by Ilizarov’s method. Khirurgiia.

[54] Shevtsov V.I., Larionov A.A., Pepeliaev A.G., Petrovskaia N.V. (1998). Distraction osteosynthesis in occlusive diseases of the limb arteries. Khirurgiia.

[55] Shevtsov V.I., Shurova E.N., Shurov V.A. (1997). Functional outcomes of legs obliterative endarteritis treatment by Ilizarov’s method. Khirurgiia.

[56] Chen Y., Ding X., Zhu Y., Jia Z., Qi Y., Chen M., Lu J., Kuang X., Zhou J., Su Y. (2022). Effect of Tibial Cortex Transverse Transport in Patients with Recalcitrant Diabetic Foot Ulcers: A Prospective Multicenter Cohort Study. J. Orthop. Transl..

[57] Hu X.-X., Xiu Z.-Z., Li G.-C., Zhang J.-Y., Shu L.-J., Chen Z., Li H., Zou Q.-F., Zhou Q. (2022). Effectiveness of Transverse Tibial Bone Transport in Treatment of Diabetic Foot Ulcer: A Systematic Review and Meta-Analysis. Front. Endocrinol..

[58] Luxon A., Syziu A., Harrison W.D., Islam A., Mason L. (2025). A Systematic Review on Tibial Cortex Transverse Transport in the Treatment of Ischemic Ulcers of the Lower Limb. Foot Ankle Int..

[59] Chang S., Chen W., Song H., Zhang F., Prà I.D., Armato U., Zhou J., Nie K., Yin M., Chiarini A. (2024). Iliac Crest Bone Distraction Surgery for the Treatment of Thromboangiitis Obliterans of Lower Limbs: A Cohort-Prospective Preliminary Study.

[60] Mo X., He C., Zhou J., Chen W., Nie K., Wei Z., Chang S. (2024). Preliminary Application of Ulnar Cortex Transverse Transport Technique in Treatment of Upper Extremity Thromboangiitis Obliterans. Zhongguo Xiu Fu Chong Jian Wai Ke Za Zhi.

