# Supplementary material for: Efficacy of the Tibial Transverse Bone Transport Technique in the Management of Thromboangiitis Obliterans: A Systematic Review
Source: J Clin Med. 2026 Jun 11;15(12):4521. doi: 10.3390/jcm15124521 (PMC13301970; doi:10.3390/jcm15124521)
Supplement: Supplementary file 1 [file jcm-15-04521-s001.zip › jcm-4344509-supplementary.pdf]

# Supplementary Material S1. Detailed PubMed Search Strategy

Search final date: 31 December 2025

Database searched: PubMed

Search strategy:

("Thromboangiitis obliterans" OR "Buerger disease")

AND

("tibial transverse transport" OR "transverse tibial transport" OR "transverse tibial corticotomy" OR "bone transport" OR "Ilizarov's technique" OR "tibial cortex transverse transport" OR "cortex transverse transport" OR "distraction histogenesis" OR "distraction osteogenesis")

Filters applied: None

Language restrictions during search: None

Gray literature search:
Gray literature was explored through manual screening of reference lists from eligible studies, citation tracking, and supplementary searches using Google Scholar to identify potentially relevant studies not indexed in the primary databases.

Screening process:
After duplicate removal, title and abstract screening were independently performed by two reviewers (R.S. and M.R.A.). Potentially eligible articles subsequently underwent independent full-text assessment by the same reviewers. Disagreements were resolved through discussion and consensus.

Eligibility restrictions applied during screening:

- Peer-reviewed clinical studies
- Human studies
- Adult patients (≥18 years)
- Accessible full-text articles
